# Complete mitochondrial genome of the hydrothermal vent barnacle *Eochionelasmus ohtai* (Cirripedia, Thoracica)

**DOI:** 10.1080/23802359.2017.1419089

**Published:** 2017-12-22

**Authors:** Se-Joo Kim, Won-Kyung Lee, Ryeo-Ok Kim, Se-Jong Ju

**Affiliations:** a Korean Bioinformation Center, Korea Research Institute Bioscience and Biotechnology, Daejeon, Korea;; b Deep-sea and Seabed Mineral Resources Research Center, Korea Institute of Ocean Science & Technology, Ansan, Korea;; c Department of Life Science, College of Natural Sciences, Sangmyung University, Seoul, Korea;; d Department of Marine Biology, University of Science & Technology, Daejeon, Korea

**Keywords:** *Eochionelasmus ohtai*, hydrothermal vent barnacle, mitochondrial genome, Fiji Basin

## Abstract

Thoracican barnacles are common in hydrothermal vent fields. Here, we characterized the first mitogenome of a hydrothermal vent barnacle. The mitogenome of *Eochionelasmus ohtai* was 15,585 bp in length and had the typical pancrustacean gene arrangement. Its protein-coding genes (PCGs) were very similar in terms of length, AT content, and start and stop codons to those of other thoracican species. The phylogenetic tree constructed with 13 PCGs divided balanomorph barnacles, including *E*. *ohtai*, into two clades. This will further our understanding of the evolution of hydrothermal vent barnacles using mitogenomes, although further mitogenomic analysis of undetermined taxa is required.

Thoracican barnacles are common in hydrothermal vent field worldwide (Herrera et al. 2015). Although 20 complete mitochondrial genomes (mitogenomes) of thoracican barnacles were registered in GenBank as of 14 September 2017, no mitogenomes of hydrothermal vent barnacles have been reported. *Eochionelasmus ohtai* Yamaguchi, 1990, comprises two proposed subspecies, *E*. *ohtai ohtai* and *E*. *ohtai manusensis*, and is common near hydrothermal vents in the Pacific Ocean (Yamaguchi and Newman 1997). To understand hydrothermal vent barnacles at a genomic level, the mitogenome of *E*. *ohtai* was investigated.

In November 2016, *E*. *ohtai* specimens were collected from hydrothermal vent fields in the North Fiji Basin (18°49′S and 173°30′W; 2722 m in depth), in the Southwestern Pacific Ocean using ROV (ROPOS, Canadian Scientific Submersible Facility). Genomic DNA extraction, mitochondrial DNA amplification, library construction and sequencing were performed according to the method of Kim et al. (2016, 2017). A complete mitochondrial genome was obtained with the software NOVOplasty 2.4 (Dierckxsens et al. 2017) and Geneious (Biomatters, Auckland, New Zealand). An ambiguous mixed peak Y at nucleotide site 4297 was changed to T by Sanger sequencing (forward primer, Eohtai + F: TCGCATATTCTACAACTTCGTC; reverse primer, Eohtai + R: TTATGATAAGGGCCATCCTG). The mitochondrial genes were annotated using MITOS (Bernt et al. 2013), ARWEN (Laslett and Canbäck 2008), and tRNAscan-SE (Lowe and Eddy 1997), and confirmed by a comparison of sequences among related species. The used specimen for mitogenome analysis had been deposited in the Library of Marine Samples (KIOST, Geoje, Korea; accession no. BS_MA00010131).

The complete mitogenome of *E*. *ohtai* was 15,585 bp in length (GenBank accession no. MF939636), and consisted of 13 protein-coding genes (PCGs), two rRNAs, 22 tRNAs, and a non-coding region. The gene arrangement and transcriptional polarity showed the ancestral pancrustacean pattern, except for some tRNAs. The base composition of the mitogenome had a 68.9% AT content. All of the PCGs had ATN as the start codon, except ND5 and ND4L, which had GTG. Most of the PCGs terminated with a complete stop codon (TAA or TAG), although three PCGs (COX3, ND3, and ND4) had incomplete stop codons (T–). The 16S and 12S rRNAs were 1290 bp (73.6% AT content) and 753 bp (67.2% AT content) in length, respectively. The tRNA genes ranged from 57 to 70 bp in size and had the typical cloverleaf secondary structure. A 304-bp-long non-coding region (73.0% AT content) was located between the 12S rRNA and tRNA*^Lys^* genes.

Based on the phylogenetic tree inferred from the PCGs, balanomorph barnacles were divided into two subclades (Figure 1). Previous studies recognized *Eochionelasmus ohtai* as the most primitive member of balanomorph barnacles (Yamaguchi and Newman 1997; Pérez-Losada et al. 2014; Herrera et al. 2015). However, our tree topology was not in accord with the phylogenetic trees based on nuclear and partial mitochondrial genes (Pérez-Losada et al. 2014; Herrera et al. 2015). Considering the diversity of thoracican species, further mitogenomic analysis of undetermined taxa is required to resolve the taxonomic position of hydrothermal vent barnacles.

**Figure 1.**
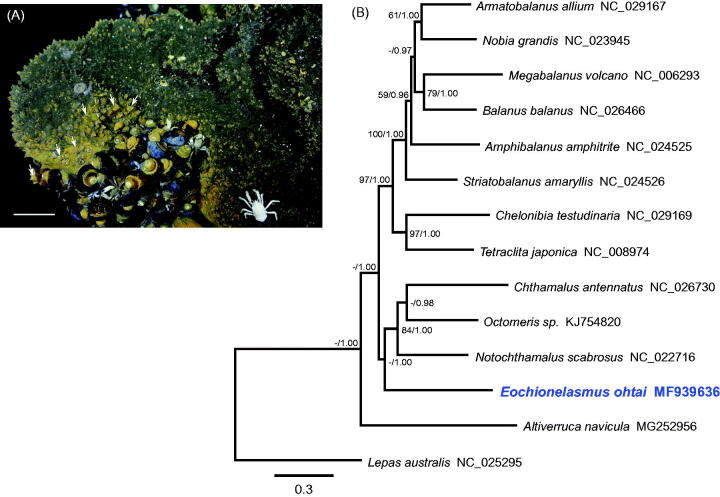
(A) Photograph of the sampling site in the North Fiji Basin where *Eochionelasmus ohtai* was collected. Arrows indicate *E. ohtai* individuals. Scale bar = 10 cm. (B) Phylogenetic tree of *E. ohtai* and related barnacles based on 13 protein-coding genes from mitogenomes. The model GTR + I+G was selected as the best evolutionary model using jModelTest 2.1.4. Numbers on internodes are the maximum likelihood bootstrap proportions (left) and Bayesian posterior probabilities (right). ‘–’ indicates bootstrap values of less than 50%.
